# Long term life dissatisfaction and subsequent major depressive disorder and poor mental health

**DOI:** 10.1186/1471-244X-11-140

**Published:** 2011-08-23

**Authors:** Teemu Rissanen, Heimo Viinamäki, Kirsi Honkalampi, Soili M Lehto, Jukka Hintikka, Tarja Saharinen, Heli Koivumaa-Honkanen

**Affiliations:** 1 Dept of Psychiatry, Kuopio University Hospital, Kuopio, Finland; 2 Dept of Psychiatry, Päijät-Häme Central Hospital, Lahti, Finland; 3 Institute of Clinical Medicine, Dept of Psychiatry, University of Oulu, Oulu, Finland; 4 Dept of Psychiatry, Lapland Hospital District, Muurola, Finland; 5 Institute of Clinical Medicine, Psychiatry, University of Eastern Finland, Kuopio, Finland; 6 Institute of Clinical Medicine, Psychiatry, University of Tampere, Tampere, Finland

**Keywords:** life satisfaction, quality of life, major depressive disorder, hopelessness.

## Abstract

**Background:**

Poor mental health, especially due to depression, is one of the main public health problems. Early indicators of poor mental health in general population are needed. This study examined the relationship between long-term life dissatisfaction and subsequent mental health, including major depressive disorder.

**Method:**

Health questionnaires were sent to a randomly selected population-based sample in 1998 and repeated in 1999 and 2001. In 2005, a clinically studied sub-sample (n = 330) was composed of subjects with (n = 161) or without (n = 169) repeatedly reported adverse mental symptoms at all three previous data collection times. Clinical symptom assessments were performed with several psychometric scales: life satisfaction (**LS**), depression (**HDRS, BDI**), hopelessness (**HS**), mental distress (**GHQ**), dissociative experiences (**DES**), and alexithymia (**TAS**). The long-term life dissatisfaction burden was calculated by summing these life satisfaction scores in 1998, 1999, 2001 and dividing the sum into tertiles. Psychiatric diagnoses were confirmed by SCID-I for DSM-IV in 2005. Logistic regression analyses were performed to assess the studied relationship.

**Results:**

The previous life dissatisfaction burden associated with adverse socio-demographic, life style and clinical factors. In adjusted logistic regression analyses, it was independently and strongly associated with subsequent major depressive disorder in 2005, even when the concurrent LS score in 2005 was included in the model. Excluding those with reported major depressive disorder in 1999 did not alter this finding.

**Limitations:**

MDD in 1999 was based on self-reports and not on structured interview and LS data in 2001-2005 was not available.

**Conclusions:**

The life satisfaction burden is significantly related to major depressive disorder and poor mental health, both in cross-sectional and longitudinal settings.

## Background

Good mental health is characterized by personal maturity, emotional and social intelligence and resilience, as well as subjective well-being [1]. Thus, life satisfaction and happiness are not only indicators of subjective well-being [1-3], but are also important dimensions of mental health [4]. It has been shown that patients with depression primarily aim at attaining indicators of good mental health, not only at reducing adverse mental symptoms [5]. Similarly, subjective well-being and life satisfaction should be the primary focus and target in health care practice [6,7].

However, in order to measure subjective well-being as a treatment outcome, reliable and easily applied measurement tools are needed. Clinically, the use of a four-item life satisfaction scale has been well accepted by patients as well as general populations [8-10]. In patient populations, it has been cross-sectionally strongly associated with various mental symptoms such as depression, hopelessness, psychosomatic symptoms, alexithymia, general psychopathology and low concurrent functional ability [8,11-13]. Among healthy general population subjects, life dissatisfaction has also been shown to predict several poor health outcomes such as psychiatric morbidity [11], depressive symptoms [14], total mortality [9], suicides [15] and fatal unintentional injury deaths [16], as well as premature work disability due to somatic and psychiatric causes [17] in follow-ups of over a decade.

At least moderate stability of life satisfaction has been reported among healthy general population subjects, and individuals with either persistently low or high life satisfaction have been found [18]. However, it is not known how persistent life dissatisfaction might relate or precede subsequently diagnosed psychiatric disorders. Thus, in order to detect long-term adverse processes resulting in poor mental health outcomes as early as possible, longitudinal research is needed on the course of life dissatisfaction with respect to psychiatric diagnoses and adverse mental symptoms in the general population. In this population-based study we investigated the role of long-term self-reported life dissatisfaction as a predictive factor for future mental health.

## Methods

The present study formed a part of the longitudinal Kuopio Depression Study (KUDEP), which was conducted in the district of Kuopio in the central-eastern part of Finland [19]. A sample of the general population (N = 3004) living in Kuopio, aged 25-64 years, was randomly selected from the National Population Register. The study protocol was approved by the Research Ethics Committee of Kuopio University Hospital and the University of Kuopio. All subjects provided written informed consent before entering the study.

A baseline study questionnaire was mailed in 1998 (**T1**) with a response rate of 68% (n = 2050). In addition, two subsequent follow-up health questionnaires were mailed in 1999 (**T2**) (n = 1722) and 2001 (**T3**) (n = 1593). A total of 1347 study subjects responded to all three data collections. In 2005 (i.e. seven years after the baseline), a sub-sample of the subjects was invited for clinical evaluation.

The inclusion criteria for the 2005 sub-sample were based on the presence or absence of self-reported adverse mental symptoms. The following cut-offs were used: **BDI-21 **score > 9, **TAS-20 **score > 57 or **LS-4 **score > 11. Half of the subjects fulfilled at least one of these criteria in all three data collection years (n = 209). A second group with a similar size and the same age and gender distribution was formed from those subjects who were asymptomatic with respect to any of these adverse symptoms during the follow-up (n = 218). The participation rate for the sub-sample was 78% (total n = 333). Out of these, three cases with inadequate data on life satisfaction were excluded. Thus, the final sub-sample comprised 330 subjects. The mean age was 56.4 years (SD = 9.6) for men (n = 142; 43%) and 55.4 years (SD = 9.5) for women (n = 188; 57%) (p = ns).

Life satisfaction was assessed with a self-reported 4-item life satisfaction scale (**LS**, range 4-20, higher scores indicating lower life satisfaction), which was originally modified from quality of life studies [20-22]. Study subjects were asked to rate their general interest and happiness in life, ease of living and experiences of loneliness with the following scores, respectively: 1 = very interesting/happy/easy/not at all lonely; 2 = fairly interesting/happy/easy; 3 = cannot say/missing data; 4 = fairly boring/unhappy/hard/lonely; 5 = very boring/unhappy/hard/lonely.

The life dissatisfaction burden, an indicator of long-term life satisfaction, was based on the sum of LS scores in the first three follow-up assessments (T1 to T3). The study subjects were divided into tertiles according to this burden: the long-term life satisfaction group^1 ^[range 12-21; n = 116 (35.2%)], intermediate group^2 ^[range 22-33; n = 107 (32.4%)] and long-term dissatisfaction group^3 ^[range 34-60; n = 107 (32.4%)].

The health questionnaires included questions concerning the socio-demographic background. Age, gender, marital status (cohabiting i.e. married or living with a partner vs. non-cohabiting i.e. unmarried or living without a partner), subjective economic status (good/fairly good vs. fairly poor/poor), subjective working ability (good vs. reduced/unable to work), current smoking (yes/no) and alcohol consumption (2-3 times per week/more vs. once a week/less) were recorded.

Depressive symptoms were rated using the self-administered 21-item Beck Depression Inventory (**BDI-21**, range 0-63) [23] and the 17-item Hamilton Depression Rating Scale (**HDRS**, range 0-52) [24] assessed by a trained nurse. The alexithymic symptoms were screened using the 20-item Toronto Alexithymia Scale (**TAS-20**; range 20-100) [25-27]. Hopelessness was assessed by the 20-item Beck Hopelessness Scale (**HS-20**; range: 0-20) [28]. The 12-item self-reported General Health Questionnaire (**GHQ-12**; range 0-36) [29] was used to assess mental distress. The 28-item self-reported Dissociative Experiences Scale (**DES-28**; range 0-100) [30] was used to assess psychological dissociation. In all of these psychometric scales, higher scores indicated higher psychopathology.

In 1999, self-reported data on previous physician-diagnosed major depressive disorder (MDD) was obtained from the health questionnaire. In 2005, the diagnoses of major depressive disorder were assessed by means of the Structured Clinical Interview for DSM-IV (SCID-I) [31,32].

### Statistical methods

Data analysis was carried out with SPSS (version 17.0). The differences between the study groups were examined with the Pearson chi-squared test for categorical variables, and analysis of variance (ANOVA) for continuous variables. In the case of variables not following a normal distribution, the non-parametric Kruskal-Wallis test was used.

Logistic regression (method:enter) was used to prospectively examine how the life satisfaction burden in 1998-2001 was related to a subsequent diagnosis of major depression disorder in 2005, and how MDD in 2005 was related to the previous long-term life satisfaction burden. In the logistic models, the LS burden was mostly treated as a continuous score, but as an outcome variable the dissatisfied group was compared with the satisfied group (LS 34-60 vs. 12-21). The final logistic regression models were adjusted for possible confounders (factors significantly associated with LS burden) and they were carried out with and without including major depressive disorder (MDD) diagnosed by a physician in 1999 in order to investigate the associations of new cases with MDD.

## Results

The socio-demographic and current clinical characteristics of the sample in 2005 according to the life satisfaction burden in 1998-2001 are summarized in Table 1. In general, subjects with long-term life satisfaction were better off than those with dissatisfaction with respect to subsequent socio-demographic and clinical factors. Long-term life dissatisfaction associated with living alone, having no children, a reduced ability to work, a poor economic and health status, current smoking and more frequent alcohol consumption. Moreover, previous long-term life dissatisfaction also associated with poorer mental health in 2005. This was the case with all of the psychometric scales (TAS, HS, GHQ, DES, LS) of the study, including both self-reported (**BDI**) and objectively assessed depression (**HDRS**) in 2005, as well as with a subsequent MDD diagnosis in 2005. Out of those with long-term life dissatisfaction, 55% (n = 59) were diagnosed with MDD in 2005, whereas among those with long-term life satisfaction the respective figure was 5% (n = 6).

**Table 1:** Socio-demographic factors and psychometric scales in 2005 according to the long-term Life Satisfaction Burden1)

Characteristics in 2005	Satisfied (LS-burden12-21) n = 116		Intermediate (LS-burden 22-33) n = 107		Dissatisfied (LS-burden 34-60) n = 107		p-value (χ^2^)
	Col%	(n)	Col%	(n)	Col%	(n)	
Male	42.2	(49)	47.7	(51)	39.3	(42)	ns

Non-Cohabiting	8.6	(10)	13.1	(14)	23.4	(25)	0.007^2)^

No child	12.1	(14)	19.6	(21)	28.0	(30)	0.011^3)^

Reduced work ability	35.3	(41)	66.4	(71)	70.1	(75)	< 0.001^4)^

Poor economic status	3.4	(4)	13.1	(14)	35.5	(38)	< 0.001^5)^

Current smoker	6.0	(7)	17.8	(19)	32.7	(35)	< 0.001^6)^

Alcohol use **≥**2/week	10.3	(12)	22.4	(24)	19.6	(21)	0.043^7)^

Major Depressive Disorder	5.2	(6)	25.2	(27)	55.1	(59)	< 0.001^8)^

	**Mean (95%CI)**		**Mean (95%CI)**		**Mean (95%CI)**		**p-value**

Age	56.6 (54.8-58.4)		56.9 (55.0-58.8)		54.0 (52.3-55.6)		0.013^9)^*

BDI	3.1 (2.5-3.7)		6.5 (5.4-7.5)		13.6 (12.0-15.3)		< 0.001^10)^*

LS	6.3 (6.0-6.6)		8.2 (7.7-8.6)		11.9 (11.2-12.6)		< 0.001^11)^*

TAS	38.2 (36.7-39.6)		46.3 (44.2-48.4)		49.9 (47.6-52.2)		< 0.001^12)^*

HS	2.5 (2.2-2.8)		4.0 (3.3-4.6)		7.7 (6.7-8.7)		< 0.001^13)^*

GHQ	8.9 (8.4-9.4)		11.8 (10.8-12.7)		15.3 (14.1-16.5)		< 0.001^14)^*

HDRS	2.8 (2.3-3.4)		5.1 (4.3-6.0)		8.0 (6.9-9.1)		< 0.001^15)^*

DES	3.3 (2.7-4.0)		5.1 (4.0-6.2)		7.9 (6.2-9.7)		< 0.001^16)^*

^1) ^Life satisfaction burden is the sum of life satisfaction scores in 1998, 1999 and 2001

^2) ^χ^2 ^= 10.0; ^3) ^χ^2 ^= 9.0; ^4) ^χ^2 ^= 33.4; ^5) ^χ^2 ^= 42.3; ^6) ^χ^2 ^= 26.3; ^7) ^χ^2 ^= 6.3; ^8) ^χ^2 ^= 69.7; ^9) ^χ^2 ^= 8.6;

^10) ^χ^2 ^= 110.5; ^11) ^χ^2 ^= 143.1; ^12) ^χ^2 ^= 61.2; ^13) ^χ^2 ^= 87.2; ^14) ^χ^2 ^= 81.3; ^15) ^χ^2 ^= 63.6; ^16) ^χ^2 ^= 28.2 All df = 2

* Kruskal-Wallis Test

BDI = Beck Depression Inventory

GHQ = General Health Questionnaire

LS = Life Satisfaction scale

HDRS = Hamilton Depression Rating Scale

TAS = Toronto Alexithymia Scale

DES = Dissociative Experiences Scale

HS = Beck Hopelessness Scale

Those with MDD in 2005 had a strongly increased OR (11.0; 95% CI 3.97-30.4) for previous long-term life dissatisfaction (LS burden scores 34-60 vs. 12-21) compared to those without MDD, even after adjustment for several concurrent socio-demographic and life style factors (Table 2). In this model, a reduced ability to work, a poor economic situation and current smoking in 2005 were also significantly associated with the previous long-term life dissatisfaction burden (model 1). When all the psychometric scales in 2005 were assessed in another model (model 2), those with low life satisfaction and hopelessness (OR = 2.13; 1.57-2.89 and 1.44; 1.10-1.90, respectively; both as continuous scores) had a significantly increased risk of having belonged to the previous long-term life dissatisfaction group (Table 2).

**Table 2:** The adjusted risks (OR 95%CI) of having belonged to those with long-term dissatisfaction (LS burden scores: 34-60 vs. 12-21)

Retrospective model 1: Major depressive disorder, health behaviour and socio-demographic variables in 2005	Age- and gender-adjusted separate OR (95% CI)	Multiple adjusted **OR (95% CI)**^**1**^
Age	0.97 (0.94-1.00)*	0.97 (0.93-1.02)

Gender	0.89 (0.52-1.53)	0.92 (0.43-1.95)

Children	2.75 (1.35-5.58)**	2.08 (0.68-6.38)

Marital Status	3.00 (1.35-6.65)**	2.04 (0.59-7.11)

**Reduced work ability**	8.43 (4.18-17.0)***	3.68 (1.56-8.68)**

**Poor economic status**	14.7 (5.00-43.1)***	6.29 (1.78-22.2)**

**Current Smoking**	7.24 (3.03-17.3)***	5.73 (1.95-16.8)**

Alcohol consumption	2.20 (1.01-4.78)*	2.49 (0.90-6.96)

**Major Depressive Disorder**	22.2 (8.89-55.5)***	11.0 (3.97-30.4)***

**Retrospective model 2: Clinical variables in 2005**	**Age- and gender-adjusted separate OR (95% CI)**	**Multiple adjusted** **OR (95% CI)**^**1**^

**Current LS**	2.51 (1.94-3.26)***	2.16 (1.58-2.96)***

**Current HS**	1.81 (1.49-2.20)***	1.41 (1.09-1.84)*

Current HDRS	1.34 (1.23-1.47)***	1.02 (0.88-1.18)

Current TAS	1.13 (1.09-1.17)***	1.02 (0.95-1.09)

Current DES	1.17 (1.09-1.26)***	1.06 (0.96-1.17)

Current GHQ	1.46 (1.3-1.6)***	1.05 (0.89-1.25)

*p < 0.05; **p < 0.01; ***p < 0.001

^1^Multiple adjusted logistic model 1 (method:enter) included the following factors: age (continuous), gender (**0 = **female; **1 = **male), children (**0 = **yes; **1 = **no), marital status (**0 = **cohabiting; **1 = **non-cohabiting), subjectively assessed reduced work ability (**0 = **no; **1 = **yes), subjectively assessed poor economic status (**0 = **no; **1 = **yes), current smoking (**0 = **no; **1 = **yes), alcohol consumption (**0 = **< 2/week; **1 = **≥2/week), current MDD (**0 = **no; **1 = **yes)

^2^Multiple adjusted logistic model 2 (method:enter) included the following factors: LS, HS, HDRS, TAS, DES, and GHQ as continuous scales

According to the retrospective final logistic model including all the above-described significant variables from models 1-2, MDD in 2005 (OR 7.79; 2.00-30.4), or life dissatisfaction in 2005 (continuous score: OR 2.12; 1.59-2.84) remained the only significant factors associating with the previous long-term life dissatisfaction burden. Excluding those who already had MDD in 1999 from the final model did not change the pattern. Hopelessness (OR = 1.35; 1.03-1.75) and life dissatisfaction (OR = 2.30; 1.67-3.17) in 2005 were significantly and MDD marginally significantly (OR = 4.67; 0.97-22.4, p = 0.054) associated with the previous LS burden.

In a prospective logistic final model adjusted for work ability, economic status, smoking, hopelessness and the LS score in 2005, the life dissatisfaction burden in 1998-2001 as a continuous score strongly predicted the subsequent MDD diagnosis in 2005 (Table 3). In this model, the significant concurrent correlates of the MDD diagnosis were the hopelessness score, smoking and a reduced ability to work, but not the LS score in 2005. When those with MDD in 1999 were excluded, the continuous LS burden still similarly predicted MDD in 2005, while smoking and reduced work ability, but not the LS score, were its significant concurrent correlates (Table 3). When the 3-category LS burden score was used instead of a continuous score in the same models, the long-term dissatisfied group (scores 34-60) had a 5-fold increased OR (5.11 (95%CI 1.72-15.2; p = 0.003) of having MDD in 2005. After excluding those with MDD in 1999, the OR of an MDD diagnosis in 2005 was 3.52 (1.10-11.3: p = 0.034).

**Table 3:** The adjusted significant risks (OR with 95% CI) of subsequent major depressive disorder according to the final prospective model with and without those who already had an MDD diagnosis in 1999 Prospective final logistic model

Variable	Multiple adjusted OR (95% CI) **Method:enter**^**1**^ all subjects	Multiple adjusted OR (95% CI) **Method:enter**^**1**^ without MDD in 1999
**LS burden**	**1.07 (1.03-1.12); p < 0,001**	**1.06 (1.01-1.11); p = 0.013**

Current LS	1.04 (0.92-1.17); p = 0.582	1.09 (0.95-1.26), p = 0.234

**Current HS**	**1.11 (1.02-1.21); p = 0.020**	1.10 (1.00-1.22); p = 0.063

**Current smoking**	**2.19 (1.08-4.45); p = 0.031**	**2.45 (1.10-5.46); p = 0.028**

Poor economic status	1.93 (0.89-4.18); p = 0.094	2.37 (0.98-5.72); p = 0.055

Reduced work ability	**2.37 (1.19-4.73); p = 0.014**	**2.32 (1.07-5.00); p = 0.032**

^1) ^Model included: LS burden (continuous scale), LS score in 2005 (continuous), HS score in 2005 (continuous), current smoking (**0 = **no; **1 = **yes), subjectively assessed poor economic status (**0 = **no; **1 = **yes), subjectively assessed reduced work ability (**0 = **no; **1 = **yes)

## Discussion

This study demonstrated that long-term life dissatisfaction burden was strongly and independently related to a subsequent MDD diagnosis and other indicators of poor mental health in a seven-year follow-up among those who had not previously reported having MDD. Multiple adjustments did not change the association.

MDD is a severe mental disorder with a strong impact on quality of life and a correlate as well as predictor of working disability [33] and functional impairment [34]. Recently, the association of positive (psychological) well-being and depressive symptoms was found in a cohort study [35]. The present study continues to highlight both the possibilities and importance of early screening. Long-term life dissatisfaction is deleterious to mental health and antecedent to MDD and might be even stronger predictor of MDD than concurrent life dissatisfaction, which has shown to correlate very strongly to depressive symptoms [14]. The main clinical importance of our results is that the process leading to psychiatric morbidity is possible to intervene early and as the main implication for clinical practice we highlight the role of primary care where screening of life satisfaction is possible during regular visits. In general, subjective well-being and life satisfaction should also be the primary focus and target of health care practice [6,7].

Previously, correlates of life dissatisfaction have included poor health behaviour [11], a poor social [16] and economic situation [8], a poor ability to work [17] and depressive symptoms [14]. The present study verifies that also longitudinally long-term life dissatisfaction is significantly related to the subsequent social and economic status, health behaviour as well as various clinical adverse outcomes. A reduced working ability, poor economic status and smoking together with MDD all indicated an independently increased probability for having belonged for several years to the long-term life dissatisfaction group. Thus, not only health care, but also other sectors of society [36] should monitor subjective well-being and life satisfaction among general population in order to facilitate mental health promotion.

The main strength of this study was its longitudinal setting, which was used both prospectively and retrospectively in order to verify the association between the life dissatisfaction burden and subsequent MDD. Also the effect of concurrent life satisfaction in 2005 and previous MDD could be investigated. No previous studies have utilized such a design. Moreover, the use of several other psychometric scales indicating poor mental health and diagnostic interviews added to the reliability and importance of the findings, even if MDD in 1999 was based on self-reports, not on the Structured Clinical Interview as in 2005.

The main limitation of the study was the lack of structured clinical interview at the beginning of the study to verify and exclude all the cases of MDD at 1998, since only the self-reported physician made diagnoses for MDD in 1999 were available. The mental health status and depressive symptoms were, however, assessed with several methods also in 1998. Another limitation of our seven-year follow-up study is the gap data on life satisfaction from 2001 to 2005, but we had that for 1998-2001 and we were investigating long-term trajectories. The current life satisfaction at 2005 was also available and it was used as an independent continuous variable in the adjusted models.

## Conclusions

The long-term life satisfaction burden is significantly related to major depressive disorder and other indicators of poor mental health in both cross-sectional and longitudinal settings.

## Competing interests

The authors declare that they have no competing interests.

## Authors' contributions

Authors RT, K-H and VH designed the study and wrote the manuscript. Authors RT and K-H managed and conducted the statistical analyses and interpreted the data. Authors HK, ST, K-H, HR and VH collected the data. All authors contributed to and have approved the final manuscript.

## Pre-publication history

The pre-publication history for this paper can be accessed here:

http://www.biomedcentral.com/1471-244X/11/140/prepub
